# Retroperitoneal Bronchogenic Cyst: MRI Findings

**DOI:** 10.1155/2013/853795

**Published:** 2013-12-04

**Authors:** R. Castro, M. I. Oliveira, T. Fernandes, A. J. Madureira

**Affiliations:** Department of Radiology, Hospital de S. João, Alameda Professor Hernâni Monteiro, 4200-319 Porto, Portugal

## Abstract

The authors describe a case of a retroperitoneal bronchogenic cyst in a 36-year-old female. She presented with abdominal pain, nausea, and vomiting. An MRI scan revealed an 8 cm cystic lesion in the left upper retroperitoneum, with intermediate signal on T2-weighted images, high signal on T1 weighted images, and lack of internal enhancement after gadolinium. After laparoscopic excision, the histology findings were compatible with a bronchogenic cyst, which is extremely uncommon in the retroperitoneum.

## 1. Introduction

Bronchogenic cysts are congenital lesions arising from abnormal budding of the embryonic foregut, during early embryogenesis. They develop most commonly in the mediastinum, posterior to the carina [1]. Retroperitoneal location is extremely rare [2]. We report a case of a retroperitoneal bronchogenic cyst, with emphasis on its MRI appearance.

## 2. Case Report

**Figure 1:**
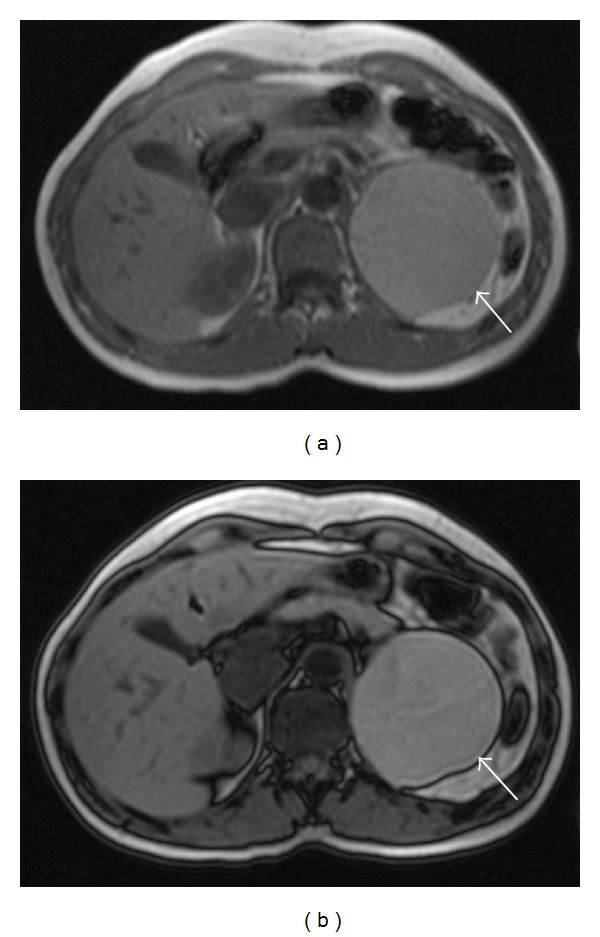
In-phase T1-weighted (a) and opposed-phase T1-WI (b) transverse images demonstrate a well-defined lesion in the left upper retroperitoneum (arrows), with high signal intensity. The lesion preserved high signal intensity on opposed-phase images, suggesting absence of microscopic fat.

**Figure 2:**
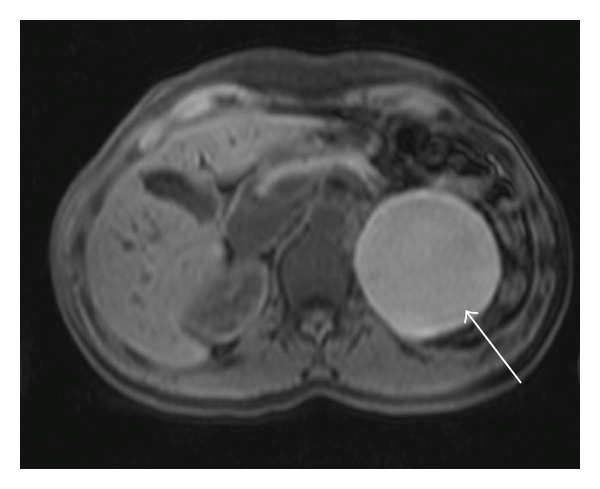
Fat suppressed T1-weighted turbo spin echo transverse image reveals high intensity in the lesion (arrow), excluding the presence of fat.

**Figure 3:**
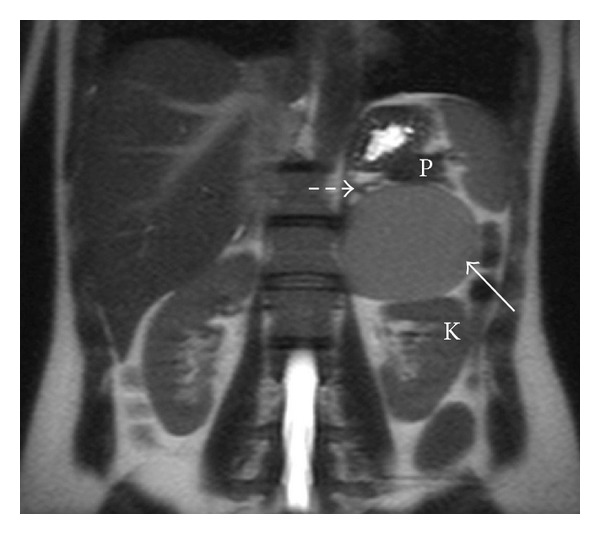
Coronal T2-weighted (TR-600 ms; TE-83 ms) MR image shows cyst contents of intermediate signal intensity (arrow). The lesion is located between the pancreatic tail (P), the left kidney (K), and the left adrenal gland (dashed arrow).

**Figure 4:**
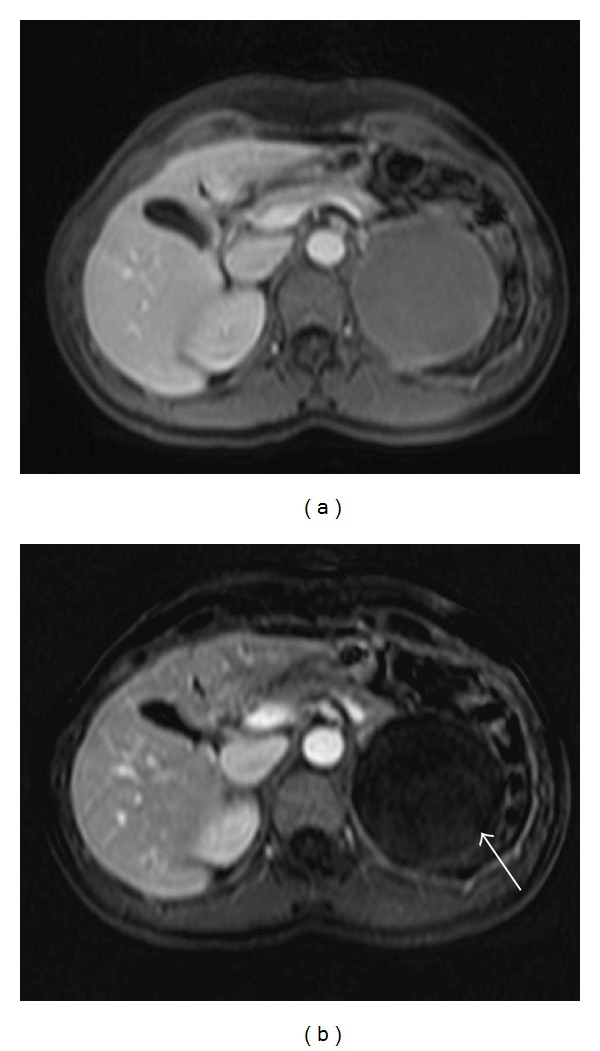
Fat suppressed T1-WI following administration of intravenous gadolinium-based contrast material (a) and subtracted image (b) show lack of enhancement.

A previously healthy 36-year-old female patient had episodes of abdominal pain in the last three months, associated with nausea and vomiting. Physical examination was unremarkable. Laboratory data showed no abnormalities: transaminases, serum amylase, and lipase were within normal values. An initial ultrasound revealed a large cystic lesion in the left upper quadrant. A contrast-enhanced abdominal MRI was performed, revealing an 8 cm retroperitoneal thin-walled cystic mass, with regular margins, located between the pancreatic tail, the upper pole of the left kidney, and the left adrenal gland. On GRE T1-weighted images (see Figure 1) it had high signal intensity, without demonstration of microscopic fat (no signal dropout in opposed-phase images). It had high signal intensity on T1-weighted images with fat suppression (see Figure 2). On T2 weighted images it revealed intermediate signal intensity (see Figure 3). After intravenous gadolinium injection, fat suppressed T1-weighted images revealed no internal enhancement (see Figure 4). No internal septations or nodules were seen.

The lesion was surgically removed via laparoscopy, and an 8 cm cystic lesion with a smooth outer surface was found in the retroperitoneum, next to the pancreatic tail.

**Figure 5:**
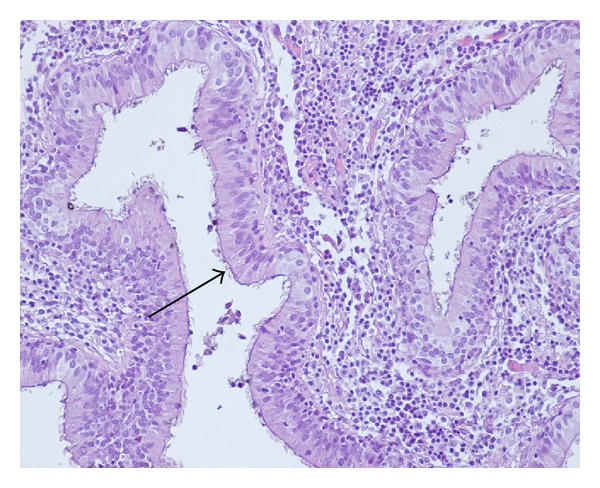
Hematoxylin and eosin stained photomicrograph (magnification ×200) shows a portion of the cyst wall, composed of pseudostratified respiratory epithelium (arrow).

Histologically, the cyst wall was covered with ciliated pseudostratified columnar epithelium (see Figure 5). In the cyst wall there were mucous glands, lymphoid tissue, and cartilage foci. These characteristics are compatible with bronchogenic cyst.

The postoperative course of the patient was uneventful and she was discharged from our institution in the seventh postoperative day, free of symptoms.

## 3. Discussion

Bronchogenic cysts are usually benign congenital lesions thought to arise from abnormal budding of the tracheobronchial analog of the primary foregut, which occurs between the 3rd and 6th weeks of gestation. If the abnormal bud retains some attachment to the primitive foregut, they are generally found near the tracheobronchial tree or oesophagus [3]. The most common locations are posterior to the carina or embedded in the pulmonary parenchyma [4, 5]. If there is complete separation of the abnormal bud, the cyst may migrate to aberrant locations, including subcutaneous tissue adjacent to the sternum, shoulder and neck, pericardium, and diaphragm. A retroperitoneal location is extremely rare, accounting for only 0.03% of all tumours [2]. Most of the retroperitoneal lesions are located near the left adrenal gland. The second most common location is the peripancreatic region [6, 7].

On pathology they are mainly cystic lesions, lined by pseudostratified columnar epithelium that rests on a connective tissue wall, containing at least one of the following: seromucous glands, smooth muscle, or hyaline cartilage [8].

Clinically, retroperitoneal bronchogenic cysts are usually asymptomatic, unless they are infected or large enough to cause compression of adjacent organs. The most common symptoms are vague abdominal discomfort and early satiety. There are described cases of pheochromocytoma-like symptoms, due to compression of the adrenal gland [9, 10], leading to increased release of catecholamines.

In the case presented the symptoms were believed to be caused by the cyst, because they subsided after the surgery.

In half of the cases these lesions are found incidentally [6]. On ultrasound they appear as anechoic lesions with or without echogenic debris. CT generally demonstrates a thin-walled, well-defined lesion, with water-density content, without enhancement. They may be hyperdense, due to proteinaceous, thick mucinous or hemorrhagic content. Fluid-fluid levels and wall calcifications may be present [11].

On MRI, variable signal intensity has been described, probably due to a mixture of water and proteinaceous fluid. Most of the cysts are isointense or hyperintense to skeletal muscle on T1-weighted images. Generally they demonstrate high signal on T2 weighted images [11, 12]. The signal intensity of the cyst contents is not suppressed on T1-weighted fat suppressed images, excluding the diagnosis of teratoma.

On gadolinium enhanced T1-weighted images, there is enhancement of the cyst wall, aiding the delineation of the thin wall [5].

The differential diagnosis of a retroperitoneal cystic lesion is large, including, among others, pancreatic pseudocyst, adrenal cyst, cystic lymphangioma, and teratoma.

The treatment of bronchogenic cyst is surgical and the prognosis is excellent [13, 14].

## 4. Conclusion

Retroperitoneal bronchogenic cyst is a very rare entity. However, it should be included in the differential diagnosis of a retroperitoneal cystic lesion, especially if it is found in the left upper quadrant.
